# ﻿Replacement names for two species of *Orthacanthus* Agassiz, 1843 (Chondrichthyes, Xenacanthiformes), and discussion of *Giebelodus* Whitley, 1940, replacement name for *Chilodus* Giebel, 1848 (Chondrichthyes, Xenacanthiformes), preoccupied by *Chilodus* Müller & Troschel, 1844 (Actinopterygii, Characiformes)

**DOI:** 10.3897/zookeys.1188.108571

**Published:** 2024-01-08

**Authors:** Loren E. Babcock

**Affiliations:** 1 School of Earth Sciences, Orton Geological Museum, The Ohio State University, Columbus, OH, 43210, USA The Ohio State University Columbus United States of America

**Keywords:** Carboniferous, Chilodontidae, headstander, junior homonym, Orthacanthidae, shark

## Abstract

Three species assigned to the same nominal genus of Paleozoic xenacanthiform shark have been combined with the name *Orthacanthusgracilis* (Chondrichthyes, Xenacanthiformes, Orthacanthidae). *Orthacanthusgracilis* (Giebel, 1848), which was originally combined as *Chilodusgracilis* Giebel, 1848, is the senior synonym; it has priority over both *Orthacanthusgracilis* (Newberry, 1857), which was originally combined as *Diplodusgracilis* Newberry, 1857, and *Orthacanthusgracilis* Newberry, 1875a. Proposed species-group replacement names are *Orthacanthuslintonensis***nom. nov.** for *O.gracilis* (Newberry, 1857) and *Orthacanthusadamas***nom. nov.** for *O.gracilis* Newberry, 1875a. *Chilodusgracilis* Giebel, 1848 is designated as the type species of *Chilodus* Giebel, 1848; this species becomes the type species for *Giebelodus* Whitley, 1940, which is a replacement name for *Chilodus* Giebel, 1848 (preoccupied by *Chilodus* Müller & Troschel, 1844, Actinopterygii). *Giebelodus* Whitley, 1940 is a junior subjective synonym of *Orthacanthus* Agassiz, 1843.

## ﻿Introduction

Three species of xenacanthiform sharks described from Carboniferous strata have been assigned to the same nominal genus and combined with the name *Orthacanthusgracilis* (Chondrichthyes, Xenacanthiformes, Orthacanthidae), either originally or subsequently. The basionym of the senior synonym, in its original combination, *Chilodusgracilis* Giebel, 1848, is homonymous with the name of an extant species of characiform fish, *Chilodusgracilis* Isbrücker & Nijssen, 1988 (Actinopterygii, Characiformes, Chilodontidae). *Chilodus* is a genus-group name that was proposed for two different nominal genera. One is a genus of characiform fish (Müller and Troschel 1844: 85–86) and the other is a genus of extinct xenacanthiform shark (Giebel 1848: 352).

The purpose of this paper is to clarify, detangle, and stabilize the nomenclature of these genus-group and species-group names.

## ﻿Nomenclatural history

**Figure 1.**
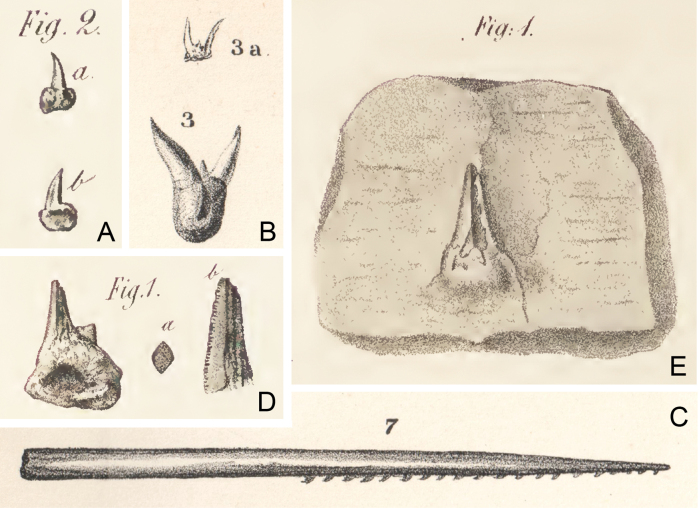
Original 19^th^ century figures of Carboniferous-age xenacanthiform shark fossils from Saxony-Anhalt, Germany, and Ohio, USA **A***Orthacanthusgracilis* (Giebel, 1848), tooth, holotype (Geiseltalmuseum Halle, GTM 1095), two views; reproduced from Giebel (1849: pl. XXIX, fig. 2a, b), 7.7 mm long. Wettin-Schichten, Wettin/Saalegebiet, Saxony-Anhalt, Germany **B***Orthacanthuslintonensis* nom. nov., replacement name for *Diplodusgracilis* Newberry, 1857a, two teeth, syntypes (repository unknown); reproduced from Newberry (1875a: pl. LVIII, figs. 3, 3a), ca 5 and 13 mm long. Upper Freeport Coal, Allegheny Group, Diamond Coal Mine, Linton, Ohio, USA **C***Orthacanthusadamas* nom. nov., replacement name for *Orthacanthusgracilis* Newberry, 1875a, dorsal spine, illustration is a composite based on syntypes (Orton Geological Museum, OSU 4467A, 4467B); reproduced from Newberry (1875a, pl. LIX, fig. 7), ca 71 mm long. Upper Freeport Coal, Allegheny Group, Diamond Coal Mine, Linton, Ohio, USA **D***Orthacanthuscarbonarius* (Germar, 1844), two teeth, syntypes (repository unknown) of *Chilodustuberosus* Giebel, 1848; reproduction of Giebel (1849: pl. XXIX, figs. 1, 1a, 1b as), length unknown. Wettin-Löbejun, Saxony-Anhalt, Germany **E***Orthacanthuscarbonarius* (Germar, 1844), tooth, syntype (repository unknown) of *Lamnacarbonaria* Germar, 1844; reproduced from Germar (1844: pl. 1, fig. 1), ca 20 mm long. Presumably from Saxony-Anhalt, Germany.

Species-group names of fossil xenacanthiform sharks that have been combined as *Orthacanthusgracilis* are as follows:

1. *Chilodusgracilis* Giebel, 1848 (Fig. 1A), reassigned to *Orthacanthus* Agassiz, 1843 by Boy and Martens (1991) and Hampe (1994, 2003). According to Articles 23.3.5, 52, 57, and 60.3 of the International Code of Zoological Nomenclature (International Commission on Zoological Nomenclature 2000), this species has priority over two species named by Newberry (1857, 1875a) (see below) that have the name *Orthacanthusgracilis* originally or after recombination.
2. *Diplodusgracilis* Newberry, 1857 (Fig. 1B), reassigned to *Orthacanthus* by Hampe (1994, 2003). It is a junior secondary homonym of *Orthacanthusgracilis* (Giebel, 1848) when both species are treated as valid species of *Orthacanthus* Agassiz, 1843 (Hampe 1994: 56–63). To remove the homonymy, the name *Orthacanthuslintonensis* nom. nov. is proposed as a new replacement name for *Diplodusgracilis* (Newberry, 1857).
3. *Orthacanthusgracilis* Newberry, 1875a (Fig. 1C). This species is a junior secondary homonym of *Chilodusgracilis* Giebel, 1848 when *C.gracilis* Giebel, 1848 is placed in *Orthacanthus* Agassiz, 1843 (Hampe 1988, 1994, 2003; Boy and Martens 1991: figs 1, 8). To remove the homonymy, the name *Orthacanthusadamas* nom. nov. is proposed as a new replacement name for *Orthacanthusgracilis* Newberry, 1875a.

Proposals of *Chilodus* as a genus-group name are as follows:

1. *Chilodus* Müller & Troschel, 1844 was erected for an extant characiform fish with *Chiloduspunctatus* Müller & Troschel, 1844 (Actinopterygii, Characiformes, Chilodontidae) as the type species, by monotypy.
2. *Chilodus* Giebel, 1848 was erected for an extinct Paleozoic xenacanthiform shark (Chondrichthyes, Xenacanthiformes, Orthacanthidae), embracing two species, *Chilodustuberosus* Giebel, 1848 (Fig. 1D) and *Chilodusgracilis* Giebel, 1848 (Fig. 1A).

The type species of *Chilodus* Giebel, 1848, designated here for nomenclatural stability, is *Chilodusgracilis* Giebel, 1848. It is the best-known species and the only one that Giebel (1848) included in *Chilodus* that is represented by a known, existing type specimen (Hampe 1994; Fig. 1A). Designation of this species as the type species follows Recommendation 69A of the Code (International Subcommission on Zoological Nomenclature 2000). The other species originally included in *Chilodus* Giebel, 1848, *C.tuberosus* Giebel, 1848, was synonymized by Giebel (1849) with *Lamnacarbonaria* Germar, 1844 (Fig. 1E); but see Romanovski (1857), who retained the combination *C.tuberosus* Giebel, 1848. Here, *L.carbonaria*, including *C.tuberosus* as a junior subjective synonym, is recombined as *Orthacanthuscarbonarius* (Germar, 1844).

Whitley (1940: 243) proposed the name *Giebelodus* as a replacement name for *Chilodus* Giebel, 1848 because the genus-group name is preoccupied by *Chilodus* Müller & Troschel, 1844. Following Article 67.8 of the Code (International Commission on Zoological Nomenclature 2000), *C.gracilis* Giebel, 1848 automatically becomes the type species of *Giebelodus* Whitley, 1940.

*Chilodusgracilis* Giebel, 1848 is here assigned to *Orthacanthus*, and *Giebelodus* Whitley, 1940 is thus a junior subjective synonym of *Orthacanthus* Agassiz, 1843.

Uses of the combination *Chilodusgracilis* are as follows:

1. *Chilodusgracilis* Giebel, 1848 (Chondrichthyes, Xenacanthiformes, Orthacanthidae), a fossil shark described from the Carboniferous of Germany.
2. *Chilodusgracilis* Isbrücker & Nijssen, 1988 (Actinopterygii, Characiformes, Chilodontidae), an extant freshwater characiform fish also known as the graceful headstander, described from Trovão, Río Aaupés, Amazonas, Brazil.

*Chilodusgracilis* Isbrücker & Nijssen, 1988 is not a junior homonym of *C.gracilis* Giebel, 1848 because, according to the exception in Art. 57.8 of the Code, and the related example, homonymy between identical species-group names in combination with homonymous generic names having the same spelling but established for different nominal genera is to be disregarded (International Commission on Zoological Nomenclature 2000).

## ﻿Systematics

**Class Chondrichthyes Huxley, 1880**

**Subclass Elasmobranchii Bonaparte, 1838**

**Superorder Euselachii Hay, 1902**

**Order Xenacanthiformes Berg, 1955**

### ﻿Family Orthacanthidae Heyler & Poplin, 1990 (see van der Laan 2018)

**Genus *Orthacanthus* Agassiz, 1843**

#### ﻿ Orthacanthus gracilis

Taxon classificationAnimaliaPoalesPoaceae

(Giebel, 1848)

B0138033-8687-5656-B150-CFD3D9935F9A

Fig. 1A

Chilodus gracilis Giebel, 1848: 352–353.
Chilodus gracilis : Giebel 1849: 70, pl. XXIX, fig. 2.
Pleuracanthus sp.: Gocht 1955: pl. VIII, fig. 5.
Orthacanthus -Typ UG: Schneider 1985: 91–92, fig. 2.
Orthacanthus carbonarius (Germar, 1844): Schneider 1988: pl. 1, fig. 4.
Orthacanthus gracilis (Giebel, 1848): Boy and Martens 1991: figs 1, 8.
Orthacanthus gracilis : Hampe 1994: 56–63, figs 1–5.
Orthacanthus gracilis : Hampe 2003: 209–210.

##### Holotype.

Tooth; Geiseltalmuseum Halle, GTM 1095, previously illustrated by Giebel (1849: pl. XXIX, fig. 2) and Hampe (1994: Fig. 1a–c).

##### Type locality.

Slate of the Wettin-Schichten (Carboniferous) from Wettin, north of Halle, Saale area, Saxony-Anhalt, Germany.

##### Remarks.

The basionym *Chilodusgracilis* Giebel, 1848 is designated herein as the type species of *Chilodus* Giebel, 1848. Whitley (1940: 243) proposed *Giebelodus* as a replacement name for *Chilodus* Giebel, 1848 (preoccupied by *Chilodus* Müller & Troschel, 1844), and *C.gracilis* Giebel, 1848 is thus the type species of *Giebelodus*. Following Boy and Martens (1991) and Hampe (1994, 2003), *Giebelodusgracilis* (Giebel, 1848), which is known only from teeth, is referred to the genus *Orthacanthus* Agassiz, 1843.

#### ﻿ Orthacanthus lintonensis nom. nov.

Taxon classificationAnimaliaPoalesPoaceae

3E7079C0-A0BB-5C5F-86A1-85D69F2D520E

Fig. 1B

Diplodus gracilis Newberry, 1857: 99.
Diplodus gracilis : Newberry 1873: 334–336.
Diplodus gracilis : Newberry 1874: 330–331.
Diplodus gracilis : Newberry 1875a: 45, pl. LVIII, figs 3, 3a.
Diplodus gracilis : Newberry 1875b: 45, pl. LVIII, figs 3, 3a.
Xenacanthus gracilis (Newberry, 1857): Olson 1946: 290–291.
Xenacanthus compressus (Newberry, 1857): Hotton 1952: 496, 499.
Orthacanthus compressus (Newberry, 1857): Hook and Baird 1986: table 2.
Orthacanthus gracilis (Newberry, 1857): Hampe 1988: 292.
Orthacanthus compressus : Hook and Baird 1988: table 1.
Orthacanthus gracilis : Hampe 1994: 63.
Orthacanthus compressus : Johnson 1999: 243–245.
Orthacanthus gracilis : Hampe 2003: 209–210.

##### Syntypes.

Teeth, repository unknown, previously illustrated by Newberry (1875a: 45, pl. LVIII, figs 3, 3a; 1875b:45, pl. LVIII, figs 3, 3a).

##### Type locality.

Upper Freeport Coal (Carboniferous), from the Diamond Coal Mine, Linton, Jefferson County, Ohio, USA.

##### Etymology.

The species refers to Linton, Ohio, the type locality.

##### Remarks.

The new species-group name *Orthacanthuslintonensis* nom. nov. replaces *Diplodusgracilis* Newberry, 1857, which after recombination as *Orthacanthusgracilis* (Newberry, 1857) is a junior secondary homonym of *Orthacanthusgracilis* (Giebel, 1848).

Detailed study of xenacanthiform materials from the Linton Lagerstätte is needed, and the type specimens need to be re-examined. Much of the systematic work on fish taxa described from Linton after 1900 has involved non-type specimens. Indeed, most published illustrations of Linton fish types are line-art drawings (e.g. Newberry 1873, 1874, 1875a, 1875b; herein, Fig. 1B, C), often with generous “restoration;” few of the types, even the ones whose repositories are known, have been photographically illustrated. Pending restudy of the type specimens of xenacanthiform sharks from the Linton Lagerstätte, *O.lintonensis* nom. nov. is proposed here as an available name that can compete in priority with other names, not as a junior synonym of any other species (compare Hotton 1952; Hook and Baird 1986; Johnson 1999).

#### ﻿ Orthacanthus adamas nom. nov.

Taxon classificationAnimaliaPoalesPoaceae

BDFD6863-936D-5C9A-9E11-D8AE14C1860C

Fig. 1C

Orthacanthus gracilis Newberry, 1875a: 56–57, pl. LIX, fig. 7.
Orthacanthus gracilis : Newberry 1875b: 56–57, pl. LIX, fig. 7.
Orthacanthus gracilis : Cope 1881: 163.
Pleuracanthus (Orthacanthus) gracilis : Case 1900: 701, pl. I, fig. 4.
Orthacanthus gracilis : Morningstar 1924: 53.
Xenacanthus gracilis (Newberry, 1875a): Olson 1946: 287.
Xenacanthus gracilis : Hook and Baird 1986: 179, table 2.
Xenacanthus gracilis : Hook and Baird 1988: table 1.
Orthacanthus gracilis : Hampe 2004: 209.

##### Syntypes.

Two dorsal spines, Orton Geological Museum, The Ohio State University, Columbus, Ohio, USA (OSU) 4467A, 4467B, previously illustrated as a composite by Newberry (1875a: pl. LIX, fig. 7; 1875b: pl. LIX, fig. 7).

##### Type locality.

Upper Freeport Coal (Carboniferous), from the Diamond Coal Mine, Linton, Jefferson County, Ohio, USA.

##### Etymology.

*Adamas* (Latin, diamond), in allusion to the Diamond Coal Mine, where the species was first collected.

##### Remarks.

The new species-group name *Orthacanthusadamas* nom. nov. replaces *Orthacanthusgracilis* Newberry, 1875a, which is a junior homonym of *Orthacanthusgracilis* (Giebel, 1848). Newberry (1875a: pl. LIX, fig. 7; 1875b: pl. LIX, fig. 7) illustrated this species with a composite figure based on syntypic dorsal spines. This species should not be confused with the other xenacanthiform species from Linton bearing the species epithet *gracilis*, based on teeth, and also referred to *Orthacanthus*, as discussed above. Replacement names for both taxa will reduce potential confusion. Cope (1881) and Case (1900) extended the stratigraphic range of this species into the Permian.

## Supplementary Material

XML Treatment for Orthacanthus gracilis

XML Treatment for Orthacanthus lintonensis

XML Treatment for Orthacanthus adamas

## ﻿Additional information

### Author ORCID

Loren E. Babcock https://orcid.org/0000-0002-9324-9176

### Data availability

All of the data that support the findings of this study are available in the main text.
